# A review of the WHO strategy on traditional, complementary, and integrative medicine from the perspective of academic consortia for integrative medicine and health

**DOI:** 10.3389/fmed.2024.1395698

**Published:** 2024-06-11

**Authors:** Rogier Hoenders, Ricardo Ghelman, Caio Portella, Samantha Simmons, Amy Locke, Holger Cramer, Daniel Gallego-Perez, Miek Jong

**Affiliations:** ^1^Dutch Consortium for Integrative Care and Health, Center for Integrative Psychiatry, Lentis, Groningen, The Netherlands and Faculty of Religion, Culture and Society, University of Groningen, Groningen, Netherlands; ^2^Brazilian Academic Consortium for Integrative Health and Department of Medicine on Primary Care, Faculty of Medicine Federal University of Rio de Janeiro, Rio de Janeiro, Brazil; ^3^Brazilian Academic Consortium for Integrative Health and Universidade de São Paulo, Disciplina de Ginecologia, Departamento de Obstetrícia e Ginecologia, Faculdade de Medicina FMUSP, São Paulo, Brazil; ^4^Academic Consortium for Integrative Medicine and Health, Lake Oswego, OR, United States; ^5^Academic Consortium for Integrative Medicine and Health and Department of Family and Preventive Medicine University of Utah Health, Salt Lake City, UT, United States; ^6^Academic Consortium for Traditional & Integrative Medicine and Health, Germany and Institute of General Practice and Interprofessional Care, University Hospital Tübingen, Tübingen, Germany and Robert Bosch Center for Integrative Medicine and Health, Bosch Health Campus, Stuttgart, Germany; ^7^Physical Medicine and Rehabilitation Department University of North Carolina at Chapel Hill, Chapel Hill, NC, United States; ^8^National Research Center in Complementary and Alternative Medicine (NAFKAM), Department of Community Medicine, Faculty of Health Sciences, UiT The Arctic University of Norway, Tromsø, Norway

**Keywords:** integrative medicine, biomedicine, TCIM, T&CM, World Health Organization, policy, academic consortia for integrative medicine and health

## Abstract

Despite important progress in modern medicine, widely regarded as an indispensable foundation of healthcare in all highly advanced nations and regions, not all patients respond well to available treatments in biomedicine alone. Additionally, there are concerns about side effects of many medications and interventions, the unsustainable cost of healthcare and the low resolution of chronic non-communicable diseases and mental disorders whose incidence has risen in the last decades. Besides, the chronic stress and burnout of many healthcare professionals impairs the therapeutic relationship. These circumstances call for a change in the current paradigm and practices of biomedicine healthcare. Most of the world population (80%) uses some form of traditional, complementary, and integrative medicine (T&CM), usually alongside biomedicine. Patients seem equally satisfied with biomedicine and T&CM, but in the field of T&CM there are also many challenges, such as unsupported claims for safety and/or efficacy, contamination of herbal medicines and problems with regulation and quality standards. As biomedicine and T&CM seem to have different strengths and weaknesses, integration of both approaches may be beneficial. Indeed, WHO has repeatedly called upon member states to work on the integration of T&CM into healthcare systems. Integrative medicine (IM) is an approach that offers a paradigm for doing so. It combines the best of both worlds (biomedicine and T&CM), based on evidence for efficacy and safety, adopting a holistic personalized approach, focused on health. In the last decades academic health centers are increasingly supportive of IM, as evidenced by the foundation of national academic consortia for integrative medicine in Brazil (2017), the Netherlands (2018), and Germany (2024) besides the pioneering American consortium (1998). However, the integration process is slow and sometimes met with criticism and even hostility. The WHO T&CM strategies (2002–2005 and 2014–2023) have provided incipient guidance on the integration process, but several challenges are yet to be addressed. This policy review proposes several possible solutions, including the establishment of a global matrix of academic consortia for IM, to update and extend the WHO T&CM strategy, that is currently under review.

## Introduction

Besides biomedicine, several other systems of medicine exist around the globe, some of which already for thousands of years. However, to date, these are largely separated worlds. Several calls from the WHO (1, 2) and World Health Assembly (3, 4)—the supreme decision-making body of the WHO—guide and support the integration of these different healthcare systems. In May 2014, the World Health Assembly adopted the resolution WHA67.18 on Traditional Medicine (TM) (5). Through this resolution, Member States are encouraged to develop and implement policies and actions to strengthen the role of Traditional and Complementary Medicine (T&CM)^1^ in national healthcare in line with the objectives of the WHO Traditional Medicine strategy 2014–2023 (1). This strategy has two overall goals to support Member States in: (1) Harnessing the potential contribution of TM to health, wellness, and people-centered health care, and (2) Promoting the safe and effective use of TM by regulating, researching, and integrating TM products, practitioners, and practice into health systems, where appropriate.

Furthermore, the WHO strategy document has three main strategic objectives that lay-out the strategic directions and specific actions for the positioning of T&CM within the countries’ health systems (1):

1. To build the knowledge base for active management of T&CM through appropriate national policies.
2. To strengthen quality assurance, safety, proper use, and effectiveness of T&CM by regulating products, practices, and practitioners.
3. To promote universal health coverage by integrating T&CM services into health care service delivery and self-health care.

In the past 10 years, the 2014–2023 WHO strategy has guided the establishment of policy documents, guidelines, technical products, centers, and institutes in support of the regulation, safe use, effectiveness, and integration of T&CM. The 2019 report summarized the achievements of member states in this regard. The authors of this paper acknowledging that considerable progress has been made in many countries in these areas, recognize that a significant number of challenges remain, and new challenges have emerged. The authors represent national consortia of academic medical institutions, from the United States, Brazil, the Netherlands and Germany. Taken together these four consortia represent over 80 academic healthcare centers and over 50 universities.

### Aim of this paper

The WHO aims to release a new 10-year T&CM strategy in 2025. The aim of this paper is to (1) describe the status of academic medicine, the integration of T&CM and the role of integrative medicine (IM) in this process, (2) identify remaining challenges related to the objectives of the 2014–2023 WHO strategy document, from the perspective of four academic consortia for integrative medicine, including the establishment of a global matrix of academic consortia for IM and (3) contribute to the future WHO strategy (2025).

## Definitions and characteristics

As the field of non-conventional medicines is highly variable and because of a lack of consensus on definitions we begin this paper with definitions and characteristics.

### Definitions of (non-)conventional medicine

Many terms and definitions have been used to describe or define non-conventional forms/systems/methods of medicine/healthcare (6), which can lead to confusion. In this paper we use the terminology and definitions as depicted in Table 1.

**Table 1:** Terminology and definitions of (non-)conventional medicine.

Terminology	Definition	Examples
Bio-medicine	Biomedicine, also called modern, allopathic or conventional medicine, is defined as clinical medicine based on the principles of physiology and biochemistry (7). It is the dominant health-care service delivery system from the 19th century onward, taught in university and practiced in hospitals/health systems in most (Western) countries.	Medication, radiation, surgery, high tech interventions, divided into medical specialties
Lifestyle medicine	Lifestyle medicine is a branch of medicine which has as goal to maintain optimal health and to prevent, treat and reverse chronic illness across all life stages. The health interventions used in lifestyle medicine include evidence based behavioral strategies, while considering equity and sustainability, to enhance self-management skills for optimizing nutrition, sleep hygiene, stress management, social connection, sexual health and fertility, physical activity and minimizing substance use and environmental exposures (8) (https://www.eulm.org/what-is-lifestyle-medicine).	Exercise, diet, relaxation, yoga, breathing, sleep mindfulness
Traditional medicine	Traditional medicine has a long history. It is the sum total of the knowledge, skill, and practices based on the theories, beliefs, and experiences indigenous to different cultures, whether explicable or not, used in the maintenance of health as well as in the prevention, diagnosis, improvement or treatment of physical and mental illness (1).	Traditional Chinese Medicine Unani Ayurveda
Complementary and alternative medicine	Complementary and alternative medicine (CAM) refers to a broad set of health care practices that are not part of that country’s own tradition or conventional medicine and are not fully integrated into the dominant health-care system. It is used interchangeably with traditional medicine in some countries (1). While alternative medicine is used instead of biomedicine, complementary medicine is used in addition to it. It should be noted that the same services and practices may be considered “Traditional Medicine” when practiced in the country of origin and “CM” when used outside the country of origin.	Herbal Medicine Acupuncture Mind–body Medicine Anthroposophic medicine Homeopathy Ayurveda outside India, Chinese Medicine outside China
Integrative medicine	Integrative medicine reaffirms the importance of the relationship between practitioner and patient, focuses on the whole person, is informed by evidence, and makes use of all appropriate therapeutic and lifestyle approaches, healthcare professionals and disciplines to achieve optimal health and healing (9) (https://imconsortium.org/about/introduction/).	All treatment modalities which are safe and effective
Integrative health or Traditional, Complementary and integrative health (TCIH)	Integrative health is a state of well-being in body, mind and spirit that reflects aspects of the individual, community, and population. It is affected by: (1) individual biological factors and behaviors, social values, and public policy, (2) the physical, social, and economic environments, and (3) an integrative healthcare system that involves the active participation of the individual and the healthcare tam in applying a broad spectrum of preventive and therapeutic approaches. Integrative health encourages individuals, social groups, and communities to develop ways of living that promote meaning, resilience and wellbeing across the life course (10).	

### Characteristics of (non-)conventional medicine

To clarify the differences between biomedicine, lifestyle, traditional, complementary, and IM, it is important to place these within the contextual framework of health care interventions as shown in Table 2: mechanism (is the proposed working mechanism of the treatment plausible from modern scientific perspective?), effectiveness (how much evidence is there for its effectiveness?) and acceptance (to what degree are these treatments accepted and implemented in health care?) (11). Acceptability to patients varies depending on the population but is important to ascertain. Patient centered care with shared decision making is critical to an effective intervention.

**Table 2:** Characteristics of (non-)conventional medicine.

	Conventional medicine	Non-conventional medicines		
	Biomedicine	Lifestyle medicine	Complementary medicine	Traditional medicine
Working mechanism	In accordance to modern science	In accordance to modern science	Not (always) in accordance to modern science	Not (always) in accordance to modern science
Evidence for effectiveness	Generally well documented	Moderate and increasing	Varying levels, but generally low or not yet tested	Varying levels, but generally low or not yet tested
Acceptance by conventional healthcare providers	High	Partial and increasing	Mostly low	Low

Biomedicine’s efficacy and mechanisms are obviously most researched and accepted. Hypotheses on mechanisms follow generally accepted modern concepts of science and medicine. Most clinicians agree that treatments should be evidence-based (e.g., established efficacy in at least two high quality randomized clinical trials; RCTs), and many of the biomedicine treatments are, although certainly not all of them (12–14).

Research shows that lifestyle medicine is insufficiently appreciated, taught, and utilized in health care, even though there is growing evidence for its efficacy (15–17).^2^ A lifestyle program consisting of diet / nutrition, movement and relaxation has been proven effective for reversal of coronary heart disease and early-stage prostate cancer (18, 19). Lifestyle interventions have shown to be a safe and cost-effective measure to reduce the risk of progression to type 2 diabetes (20), and to effectively lowering blood pressure in patients with hypertension (21). Promoting lifestyle changes is also an effective intervention for mental health (22). Several studies have shown improvements in overall (mental) health and reduced relapse risk upon lifestyle interventions (22–24). Furthermore, a systematic review on lifestyle interventions adapted to persons with serious mental illness has reported on promising reductions in weight loss and reduction of some risk factors for metabolic syndrome (25). Lifestyle medicine is still underutilized in conventional health care settings, although acceptance is growing (26).

It is argued that T&CM often presents weak or conflicting evidence of effectiveness (27). Possibly not all available evidence is well known. In 2019, at the initiative of the Latin American and Caribbean Center for Health Sciences Information of PAHO/WHO (BIREME/PAHO/WHO), an official Virtual Health Library (VHL) specialized in MTCI was created to gather and systematize evidence in the area (28).^3^ There are currently more than 1.5 million bibliographic references on this platform and more than 2,000 systematic reviews grouped into 28 evidence maps that assess the quality of studies and the effect of more than 300 specific interventions on clinical outcomes, e.g., depression, anxiety, quality of life, stress, quality of sleep, pain relief, hypertension, diabetes, and cancer. The T&CM evidence maps prepared in collaboration with the Brazilian Academic Consortium for Integrative Health are all available in open access (29).

Besides concerns about its effectiveness, the proposed mechanisms of action of some of these modalities (for example homeopathy or Reiki) are often based on theories that are not part of modern concepts in science and biomedicine (30, 31). These medicines are therefore generally not accepted and not applied in biomedical healthcare. However, some T&CMs (32, 33) are brought closer to acceptance by contemporary science through rigorous research on effectiveness and underlying mechanisms involved. For example, acupuncture appears to be effective in the treatment of non-specific low back pain (34) and the underlying analgesic mechanism of how acupuncture can modulate the nervous system and pain pathways to alleviate pain have been demonstrated (32, 33, 35–37). Likewise, yoga seems to be effective, e.g., for back pain (38) or cancer-related symptoms (39), and plausible biological mechanisms for these effects have been proposed (40). There is some evidence for dietary supplements such as omega-3 fatty acids (41) and folate (42) for mental disorders. The efficacy of herbs such as St. John’s wort (*Hypericum perforatum*) for depression (43) and Ginkgo (*Ginkgo biloba*) for mild cognitive impairment and dementia (44) have been demonstrated. The supplement melatonin has proven efficacy for sleep disorders (45), and probiotics have been shown to have a beneficial and effective role in the prevention and treatment of several diseases including diarrhea (46) and irritable bowel syndrome (47). There also appears to be some evidence for multimodal naturopathic medicine as a complex intervention for cardiovascular disease, musculoskeletal pain, type 2 diabetes, polycystic ovary syndrome, depression, anxiety, and other chronic conditions (27, 41).

Specific modalities do not necessarily stay in one of the categories as depicted in Table 2. They can move from traditional to complementary and even to biomedicine when evidence on their effectiveness and mode of action emerges. In the case of mindfulness-based interventions, several high-quality studies have shown that programs such as Mindfulness-Based Stress Reduction led to clinically relevant improvements in outcomes in several conditions and disorders and are being accepted and implemented to a greater extent within the health care system (48). Specific modalities can also move into the other direction when evidence emerges that they do not work. For example, research does not support the often-heard claim that high-dose oral vitamin C supplements boosts the immunity and decreases the risk of respiratory infections in the general population (49).

## Status of academic medicine

Medicine as it is taught in universities and most practiced in academic hospitals, often referred to as biomedicine, is the dominant healthcare system in the (Western) world. This position is not only explained by scientific advancement. It is underscored by social, cultural, economic and political conditions of biomedical knowledge construction (50). Biomedicine has made incredible progress in the molecular and genetic understanding of disease, in high-tech innovations and in available treatments. A lot of suffering has been prevented and many diseases cured (51, 52).

However, not all patients respond well to available treatments. Interventions tend to be fragmented, focused narrowly on single organ systems. Beyond health screenings and vaccination, most care occurs after a patient has become ill. Episodic care can be transactional, poorly coordinated and conducted over many brief visits. It may therefore frequently incumbent upon the patient to coordinate and initiate care. Biomedical health care also often lacks strong tie to public health and community approaches. Another challenge is that non-communicable (lifestyle related) chronic diseases have become endemic. Additionally, there are side effect and safety concerns of many medications and interventions, and the cost of healthcare continues to unsustainably soar. Many healthcare professionals suffer from chronic stress and burn-out in a strictly regulated bureaucratic system of protocols and managed care, which impairs the therapeutic relationship (53). Finally, humanity faces huge healthcare challenges, such as pandemics, shortage of skilled personnel, increase of mental disorders, climate change and war. Biomedicine alone cannot solve all these (54).

### Side effects

The incidence of adverse side effects of drugs and interventions have made iatrogenesis one of the major public health problems in developed societies, whose causes, in addition to errors and negligence, are of a systematic nature (55). Patient safety has become a major concern in health care worldwide. Of patients admitted to hospitals, 3.7 to 17.7% are inadvertently harmed by the way their health care is delivered. Avoidable adverse events lead to a greater annual loss of life than traffic accidents, AIDS, or breast cancer (56–58).

### Costs

Healthcare costs continue to escalate, taking 17 percent of gross domestic product (GDP) in the United States, 9.5 percent in Brazil, and 10 in the Netherlands (59).^4^ The increase of costs seems largely related to chronic disease and lifestyle behavior such as diet, exercise and smoking (60). Indeed, the contribution of lifestyle to modern chronic disease has been estimated at 80% (61, 62) and even 95% (63). One possible strategy to reducing costs is inviting patients to take a more active role in their recovery, for instance by applying therapeutic lifestyle changes like exercise, diet and relaxation (15, 22). There is growing evidence for the effectiveness of lifestyle changes for improving health (20, 21, 25). There is also evidence for the cost effectiveness of lifestyle changes, which can run up to 2,360 dollars per person per year (64). For every $1 invested in the truth anti-smoking campaign, the United States saved more than $6.80. This campaign decreased youth smoking by 22% from 1999 to 2002 and averted $1.9 billion in future health care costs. Every $1 spent on evidence-based programs that increase physical activity, improve nutrition, and prevent tobacco use saves $5.60 in health spending within 5 years and up to $6.20 within 10 years (65).

### Therapeutic relationship

Another concern in medicine is the quality of the therapeutic relationship, which seems threatened by managed care, focus on protocols and evidence-based medicine at the expense of patient centered approaches, and a tendency to reductionism, narrowing the view to diseases or symptoms and losing sight of the whole person in their context. The original definition of evidence-based medicine is ‘(1) the conscientious use of current best evidence in (2) making decisions about the care of individual patients or the delivery of health services, (3) taking preferences and needs of patients into account’ (66, 67). However, the last part of this definition is often neglected. This, together with a tendency toward uniformity and efficiency (in the form of guidelines, treatment protocols and clinical pathways) can lead to a ‘one size fits all approach’, which can impair the therapeutic relationship and is in clear contrast to the original idea of evidence-based medicine (66). The reduction of length of visits to the bare minimum limits the capacity to engage patients in sustainable behavior change, further driving a medication and procedural predominant practice style.

### A new paradigm

These concerns and challenges in academic medicine call for a change in the current paradigm and practices, including patient care, scientific research, education, clinician training, and policy (68, 69). Indeed, in biomedicine there is a growing awareness of the need for a multi-dimensional perspective beyond the current paradigm that is primarily focused on medication and technology (70, 71) taking the whole person into account (72). This can be observed in concepts like ‘personalized medicine’ (73–75), ‘shared decision making’ (76), ‘patient-centered care’ (77) and positive health (78).

One such new paradigm is ‘integrative medicine’, which was introduced by a consortium of Academic Health Centers in the United States in the late 1990’s in response to the before mentioned challenges in biomedicine. Currently, this IM consortium has more than 75 active academic health centers as its members (including Duke, Harvard, Stanford, Yale) (16, 79).^5^ The Consortium defines IM as: ‘the practice of medicine that (1) reaffirms the importance of the relationship between practitioner and patient, (2) focuses on the whole person, (3) is Informed by evidence making use of all appropriate therapeutic approaches, healthcare professionals and disciplines (including T&CM) to (4) achieve optimal health and healing (80).”^6^ Other national consortia for IM were founded in Brazil (2017) (81) the Netherlands (2018) (82),^7^ and Germany (2024). This third principle includes the integration of T&CM and biomedicine and may help to overcome (some of) the before mentioned challenges to biomedicine by combining their strengths and balancing their weaknesses.

### Improving the therapeutic relationship

The focus on an optimal relationship and the holistic approach of IM may increase disclosure and communication, adherence to the treatment plan, improve the therapeutic relationship (83) and enhance treatment outcome (84–86). Treatment outcome has been shown to be highly dependent on the quality of the therapeutic alliance (87–90), while a personalized approach may also improve outcome (74). IM represents a higher-order system of systems of care that emphasizes wellness and healing of the entire person (bio-psycho-socio-spiritual dimensions) as primary goals, drawing on both conventional and T&CM approaches in the context of a supportive and effective physician-patient relationship (71). This approach encourages consideration of how symptoms fit together with a particular focus on how physical and psychological health interplay.

### Reducing healthcare costs

Findings from economic modeling research suggest that combining T&CM and biomedicine may improve cost-effective long-term outcomes (91–95), although cost-effectiveness for the majority of T&CM still needs to be evaluated. Examples of interventions that were found to be cost effective compared to usual care are: acupuncture for migraine, manual therapy for neck pain, spa therapy for Parkinson’s, self-administered stress management for cancer patients undergoing chemotherapy, pre- and post-operative oral nutritional supplementation for lower gastrointestinal tract surgery, biofeedback for patients with “functional” disorders (e.g., irritable bowel syndrome), and guided imagery, relaxation therapy, and potassium-rich diet for cardiac patients (91).

### Reliable information

IM stimulates scientific research on the safety and effectiveness of T&CM which could prevent false claims and increase safety by providing reliable information to the public. Currently, a collaborative project is ongoing between the Brazilian academic consortium on IM in partnership with the Latin American and Caribbean Center on Health Sciences Information of the Pan American Health Organization (PAHO) to develop evidence maps of clinical effectiveness in T&CM based on systematic reviews of clinical studies (29). Accordingly, it can be expected that the increasing academization and organization of T&CM in the sense of IM will lead to a general promotion and systemization of research activities in this field.

### Regulation

Taking T&CM use more seriously, increasing public attention and stimulating research and policy making will also improve regulation of products and therapists. This is reflected in the increasing number of WHO member states who have regulations on herbal medicine in all six regions of the world. Again, the academization of IM will lead to an increased public awareness on the importance of regulation of this field.

### Improved treatment outcomes

This could be achieved by developing novel therapies. For instance, Dr. Youyou Tu was awarded the Nobel Prize in 2015 for the discovery of antimalarial properties of artemisinin, a herb that is part of Ayurveda and T&CM (96). Improved outcomes may be achieved by not only looking at symptoms and problems and trying to eradicate them (pathogenesis), but also at the strengths and qualities of patients and finding ways to increase them (health promotion or salutogenesis) (97). For example, it has been shown that integrative oncology, i.e., the combination of biomedical cancer therapies with T&CM, can lead to higher health-related quality of life in cancer patients than biomedicine alone (98).

### Inducing a healthy lifestyle

IM has always stressed the importance of a healthy lifestyle. Motivating and teaching patients to improve lifestyle comes with less costs and can increase self-esteem, responsibility for one’s own health and more independence from therapists (15, 22–24). It may not only reduce pathology, but it may also enhance health and wellbeing by fostering positive emotions like calmness, empathy and self-actualization (99, 100). Lifestyle medicine, as previously described, is increasingly adopted by biomedicine (101) and may as such may guide the integration process of T&CM into conventional healthcare.

Three examples of specialties in which integrative medicine has been applied successfully are:

“Integrative psychiatry (1) emphasizes the importance of the therapeutic relationship between clinician and patient using shared decision making and a personalized approach. It (2) focuses on treating the ‘whole person’ from a holistic perspective, considering mind–body and its systems as interrelated, with biological, mental, emotional, cultural, ecological and spiritual / religious aspects. It (3) seeks to provide the ‘best of both worlds’ combining biomedicine with T&CM based on evidence for their safety and efficacy. Its focus (4) is on increasing qualities and strengths (salutogenesis) as well as decreasing symptoms (pathogenesis) and it aims for increasing general wellbeing and mental health” (11).

“Integrative oncology is a patient-centered, evidence-informed field of cancer care that utilizes mind and body practices, natural products, and/or lifestyle modifications from different traditions alongside conventional cancer treatments. It aims to optimize health, quality of life, and clinical outcomes across the cancer care continuum and to empower people to prevent cancer and become active participants before, during, and beyond cancer treatment (102, 103).”

“Pediatric integrative medicine represents an evolution in pediatric care, a paradigm that embodies a philosophy consistent with long-standing holistic principles of quality medical care. It is defined by several core guiding principles: (1) Preventive: True primary care pediatrics is proactive rather than reactive. Prescribing lifestyle solutions to prevent disease is generally preferable to costly and potentially risky treatments. Lifestyle prescriptions may include food, activity, nature, creativity, rest, mindfulness, and connection with others. (2) Context-centered: Children must be nurtured within the context of healthy families, social context, and schools. Health in mind, body, and spirit depends on how suitable the environment is for the child. (3) Relationship-based: Only through open communication and building trust are we best able to work together to ensure each child’s optimal health. The connection between health professionals and families has its own healing potential. (4) Personalized: Health is not a one-size-fits-all proposition. Each child carries a unique potential based on a complex interplay of genetic and environmental factors. There is no medical treatment that can be guaranteed as safe for 100% of any population. Each family has the inherent right to make healthcare decisions for their children, keeping in mind the best interests of the child as well as legitimate public health concerns that ethically inform these decisions. (5) Participatory: Creating health should be a collaborative process, actively encouraging participation and putting children and families back in control of their own health. Patient-centered care creates hope and empowers families to make sustainable changes, inspiring children to create the future they deserve. (6) Ecologically sustainable: how we practice healthcare affects the environment, which has a measurable and cyclical impact on our health. The health and well-being of all the Earth’s inhabitants are intimately tied to the health of our planet. (7) Evidence-informed: Therapies that are evidence-informed while using the safety-effectiveness rubric are considered as part of the treatment plan (52, 104).”

Despite these successful initiatives, academic medicine frequently regards T&CM with criticism, dismay, and distrust. In many countries biomedicine and T&CM are still largely separate worlds, sometimes with hostile attitudes toward another. However, in the last decades this seems to change. The foundation of four academic consortia for integrative medicine is an indication for this. This apparent change in openness to T&CM may be because integration is not only a current tendency in medicine, but also a trend fitting the contemporary spirit of the age in which integration seems to be the most common focus. It can be observed in religion, philosophy, spirituality and psychotherapy as well (105). It is also reflected in the WHO Traditional Medicine strategy 2014–2023.

## Assessment of the WHO traditional medicine strategy 2014–2023

The WHO Traditional Medicine strategy 2014–2023 set out ambitious goals for its member states regarding research; regulation of T&CM practices, T&CM practitioners and T&CM products; and integration of these into health systems. It also included self-care and prevention. It proposed three main strategic goals which are reviewed in the next paragraphs. Also recommendations are formulated to inform the upcoming WHO T&CM strategy.

### Strategic goal 1: current practice related to active management of T&CM through national policies

T&CM contribute to the Sustainable Development Goal (SDG-3; ‘ensure healthy lives and promote well-being for all at all ages’) of achieving Universal health Coverage (UHC) and ensuring that all people have access to care (United Nations 2019). Several World Assembly (WHA) resolutions requested the WHO to provide technical support and guidance to member states, for the integration of healthcare services (106). In European countries, T&CM are mostly used complementary to the conventional system (107). The top three reasons for T&CM use are: (1) having an expectation of benefits of T&CM, (2) dissatisfaction with conventional medicine and (3) the perceived safety of T&CM (37%) (108). Another reason reported is that T&CM resonates with one’s values or perspectives on health (108, 109). Tangkiatkumjai et al. (108) found that internal health locus of control as an influencing factor for T&CM use is more likely to be reported in Western populations, whereas the social networks is a common factor among Asian populations. Affordability, easy access to T&CM were significant factors among African populations. These factors, along with the greater availability of health information on the internet, and similar degree of satisfaction between T&CM and biomedicine, seem to be driving forces behind the growth in the use of T&CM in Western and Eastern countries in recent decades (110).

T&CM is present in almost every country in the world and the demand for its services is increasing (1). Up to 76% of the world population uses some form of T&CM each year (111). In many countries, they are the main healthcare services to the population since ancestral times (2). T&CM are an important and often underestimated health resource with many applications, especially in the prevention and management of lifestyle-related chronic diseases, and in addressing the health needs of aging populations. In an ideal world, traditional medicine would be an option provided by a well-functioning, people-centered, evidence-informed health care system that balances curative services with preventive care (2).

In the WHO’s 2019 Global Report on Traditional and Complementary Medicines (2) it is described that T&CM is an important and often underestimated health resource, specifically in the prevention and management of lifestyle-related chronic diseases, by stimulating a healthy lifestyle and in addressing the health needs of aging populations. In the light of the recent surge of healthy lifestyle interventions in biomedicine and a call for more prevention, in policy making, countries could benefit from the experience and knowledge of T&CM in these field.

Recently, the WHO has introduced the term TCIM (Traditional Complementary and Integrative Medicine) to emphasize the world-wide need to take different kinds of medicine into account. TCIM includes traditional medical systems such as Ayurveda and Traditional Chinese medicine, non-traditional and complementary ones such as Naturopathy, lifestyle medicine (nutrition, exercise, sleep), Homeopathy and Anthroposophic Medicine, and use of natural medicines such as herbs, and mind–body interventions (mindfulness, yoga), besides biomedicine (2).

Whether T&CM is fully integrated, partly integrated or not integrated in the country’s dominant health system depends on factors such as national regulations, appropriate education, funding, information, availability of services and multidisciplinary collaboration (2). Different models have been developed and described on how T&CM can be integrated into the conventional health system. They can be classified into five models ranging from coexistence, cooptative, cooperative, collaborative, to patient-centered care (112). The more coexistence and cooptative models for T&CM integration have distinct roles for different health care professionals, whereas the cooperative and collaborative models are team-based, with formalized interaction between the conventional health care professionals and T&CM practitioners (112).

At present, the practice of T&CM highly varies per country and is idiosyncratic. Such practice depends on the personal philosophies, values and clinical perspectives of its practitioners, and the goals of diverse training programs, clinics or hospitals where integrative treatment approaches are employed (113). Asian countries such as China, Japan, and the Republic of Korea have well-established systems of T&CM integration, including supportive legislation, well implemented regulatory systems for herbal medicines, T&CM practices and practitioners, as well as T&CM education systems (114). In African countries, traditional healers and natural medicines are widely used by its population, but hardly integrated into the mainstream health systems (115, 116). An illustrative example of the integration process of T&CM in a European conventional health system is that of integrative oncology in the German speaking countries. An active group of clinicians, researchers and practitioners have systematically developed a step-by-step basis for integrative oncology care in Germany and Switzerland. They have defined education competencies for oncology physicians regarding T&CM (117). As a next step, they have developed and tested a consultation framework for the training of oncology physicians to advise their patients about the effectiveness and safety of T&CM (118). Furthermore, they also have developed criteria for guiding cancer patients to find a reputable T&CM practitioner (119). Within this collaborative, they also work in parallel to further identify the needs, provide reliable information, foster communication, and support decision-making about T&CM for patients with cancer (120). In sharp contrast to the German speaking countries, in Finland there seems to be no integration of T&CM into the conventional health system. Finland lacks any regulation or guidelines on T&CM, and there is very little academic research on the subject (121). Such world-wide variety in the integration of T&CM seems undesirable, specifically from the needs and perspectives of patients.

In the past decades, the number of countries that have implemented some form of regulation on T&CM has grown. In 1999, only 25 WHO member states had a national regulatory policy on the subject, 45 had national legislation, and 65 countries had specific regulations on herbal medicine. In 2018, a total of 98 of the 194 WHO member states had a national policy, 109 had legislation, and 124 countries had regulations on herbal medicine in all six regions of the world (2). Despite this apparent increase, T&CM is still not regulated appropriately in almost half (49.5%) of the countries world-wide. Take the example of the European Union, where most T&CM modalities are practiced in a very similar way. There are European countries where T&CM or some of its modalities are regulated either as conventional medicine (e.g., chiropractic in the United Kingdom), complementary medicine (e.g., Switzerland), alternative medicine (e.g., Norway), or not regulated at all (e.g., Finland) (122). From the perspectives of its users and open border policies, it becomes apparent that this confusing and disharmonized regulation on T&CM should be addressed more prominently. An often-heard argument against T&CM regulation is that regulation may grant these non-conventional types of medicine and T&CM practitioners undue legitimacy or recognition (123). However, a recent systematic review on the subject concluded that there appears to be broad support for the regulation of T&CM, but that there is wide variation in opinions among the different stakeholders as to how this should be applied (123).

Recommendations regarding goal 1:

- The confusing and disharmonized regulation of T&CM between countries should be addressed more prominently and ultimately be harmonized.
- Strategies of how to stimulate, persuade and guide countries that still lack any kind of regulation should be developed.
- Strategies should be developed on how to make sure countries make more use of the experience and knowledge of T&CM regarding policy making in the fields of prevention, lifestyle and health promotion.

### Strategic goal 2: current practice related to the quality assurance, safety, proper use, and effectiveness of T&CM

Despite its increasing popularity, the field of T&CM is faced with many challenges, such as problems with quality standards, unsupported claims for safety and or effectiveness, challenges with sufficient research funding and appropriate research methodologies, lack of familiarity by conventional healthcare providers about evidence-based T&CM, absence of reimbursement mechanisms for T&CM, non-disclosure of T&CM use by patients, and unreliable sources of information. These challenges that T&CM is facing are described in more detail below.

#### Safety concerns

Users often associate T&CM with nature, and that all that comes from a natural source is good and safe (124). A recent study reported that as much as 90% of users regard T&CM as safe and are not aware that these natural medicines may cause unwanted side effects (108). However, the use of, for example, herbal preparations may involve some risk, potentially translating into adverse effects, interaction with other drugs, and contamination (125). For example, hepatotoxicity, allergic reactions, or gastrointestinal problems have been reported to occur after intake of herbal medicine (126, 127). Furthermore, one of the most prominent risks associated with the combined use of herbal preparations and chemotherapeutic agents in cancer treatment is that of herb-drug interactions. A systematic review revealed that six herbal preparations have potential clinically significant interactions with chemotherapeutic agents in humans (128). Contamination of herbal preparations with heavy metals may pose another serious potential risk to the health of its users. A recent study in which 1,773 samples of herbs around the world were investigated for contamination with heavy metals, almost one-third (31%) had at least one metal that was over the allowed limit according to the Pharmacopeia standards (129). The reporting of adverse effects for other T&CM modalities seems to be of lower frequency and severity as compared to herbal preparations. Nevertheless, for example minor and serious adverse effects such as organ or tissue injuries may occur upon acupuncture treatment (130). Furthermore, the prevalence of adverse effects in meditation-based therapies was found to be 8.3%, like that reported for psychotherapy practice in general (131). Most frequently reported adverse effects related to meditation practices were anxiety and depression (131). Likewise, injury risk of yoga seems to be comparable to that of other physical activity (132). A possible indirect risk with relevance for general safety is that T&CM use may cause a delay in appropriate diagnoses of patients and/or a delay in receiving effective conventional treatment (133). However, epidemiological data do not suggest that T&CM users systematically make less use of conventional medicine than non-users (134), so this supposed risk is probably limited to individual cases.

#### Lack of standardization

For most T&CM modalities, there is a large heterogeneity in how it is applied in daily practice. For example, T&CM modalities such as acupuncture (135) or yoga (136) encompass a variety of techniques and styles, and the choice for a specific technique if often based on the cultural setting in which it is practiced or practitioners’ personal experiences. The lack of standardization of T&CM practices poses a challenge for integrating the modality within the institutionalized framework of conventional healthcare. Moreover, the lack of standardized T&CM treatment protocols hampers scientific evaluation of its effectiveness and reproducibility of its results. However, standardization of T&CM seems to collide with the intrinsic principles on which it was developed: such as individualized treatment and a holistic (multidimensional) perspective on healing.

#### Claims of effectiveness

The number of scientific publications on T&CM has doubled in the last decade (137). Although the evidence for T&CM is growing, and some modalities such as mindfulness and acupuncture have found their way in clinical guidelines and recommendations (138, 139), the majority of the broad range of T&CM modalities lacks sufficient evidence to claim effectiveness (140). Therefore, misinformation about the effectiveness of certain T&CM modalities continues to spread and may create false hope among patients about the possible effects of these interventions (141). Specifically in cancer, it is known that patients are eager to try any T&CM treatment as to keep their hope for survival alive (142). Patients need to be protected from false hope, but also from false hopelessness. Therefore, it is of great importance to continue to search for and test promising T&CM modalities.

#### Research (methodology)

One of the research challenges with T&CM is lack of sufficient funding and finance. In contrast to the large pharmaceutical industry-sponsored research, there is very little industry-based research for T&CM (143, 144). This is mainly because T&CM modalities and natural products cannot be patented, and hence commercial parties that research them cannot guarantee that they will have return on investment of the research. Another challenge and highly debated area is to apply appropriate methodologies to investigate such complex and multidimensional interventions as T&CM (145, 146). Since these procedures are usually already widely used in health care without adequate clinical testing, their scientific testing often follows different guidelines than those of biomedical preparations with their clear sequence from phase I to phase IV studies (147). Additionally, the methodology, especially in the case of non-pharmacological approaches, often has to be adapted, since the usual principles like double blinding or placebo control are difficult or even impossible to realize. Other challenges to the conduct and application of T&CM research may be lack of institutional support, research training and collaboration, and diverse views of evidence (148).

#### Non-disclosure

Another challenge is that most patients (66%) do not inform their physician about their use of T&CM (109). Reasons for non-disclosure of T&CM use is lack of inquiry from the physician, fear of disapproval from their physician, perception of disclosure as unimportant, belief that the physician lacks knowledge and time on T&CM, and the belief that these modalities are safe (149). Non-disclosure of T&CM use to their treating physician is an undesirable situation since it may lead to potential health risks such as the drug-herb interactions as described above.

#### Trustworthy information

It seems urgent to facilitate the spread of reliable information on T&CM, as most users get information on these treatments through family and friends, social networks and media, and the internet (142). The quality of this information varies greatly (145, 150), and often contains non-proven claims about T&CM or promotes controversial alternative treatments. It has been reported that users wish to receive unbiased information and advice about T&CM use in open communication with their physician or other health care provider (142, 151).

#### Lack of knowledge by healthcare providers

Health care providers such as physicians and nurses seem to lack adequate training and knowledge to inform their patients about T&CM use (152–156). It is however important to note that their level of knowledge on T&CM is mostly surveyed through self-assessment. Development of instruments that directly measure their T&CM knowledge are needed (153). Regardless of how their knowledge is assessed, it has been reported that healthcare providers see a need to have better knowledge on the topic to be able to address questions that patients may have (157, 158). Introducing basic knowledge on the safety and efficacy of T&CM into the medical training of healthcare professionals seems to be an important strategy forward to increase their knowledge. Other strategies by which to increase the knowledge among conventional healthcare providers are to enhance interprofessional collaboration with T&CM providers and to facilitate their access to reliable and unbiased information on T&CM (157, 159).

Recommendations regarding goal 2:

- Map the existing evidence base and quality of that evidence for some T&CM (e.g., acupuncture, botanicals, mind body medicine), and make it accessible free of charge through thrust worthy authorities or organizations, such as WHO.
- Stimulate high quality scientific research on T&CM, specially those in high usage by the public.
- Ensure sufficient funding for research on T&CM.
- Systematically address adverse events in clinical trials as well as in clinical care.
- Develop guidelines on how to address safety specifically for T&CM.
- Invest in standardization and treatment protocols, while respecting the personalized nature of T&CM.
- Systematic control of T&CM products on possible components of endangered species (plants and animals).
- Practitioners of biomedicine and of T&CM should provide trustworthy information to the public; balancing between false hope and false hopelessness.
- Train biomedical healthcare professionals in accurate information about T&CM and in the non-judgmental addressing of T&CM during consultation.
- Develop and implement a monitoring system for the safety of T&CM practices and products similar to existing monitoring systems for medicinal products.
- Facilitate the development of guidelines and communication tools for the disclosure of (concomitant) T&CM use with recommendation by the WHO.
- Stimulate educational guidelines/curriculum for basic and general knowledge of T&CM addressed at conventional healthcare professionals.

### Strategic objective 3: promote universal health coverage by integrating T&CM services into health care service delivery and self-healthcare

#### Reimbursement of T&CM services

In many countries, T&CM are the main healthcare services to the population since ancestral times (2). Although up to 70% of the world population depends on T&CM as the first line of treatment, in many countries T&CM is still mainly offered outside the dominant health-care system. Therefore, costs associated with T&CM use are often not covered by reimbursement systems from government funding or health insurance companies. Most patients thus must pay for T&CM out of their own pocket (91, 160, 161). For the United States it was estimated that in 2007, about 14.9 billion dollar was spend out-of-pocket by US adults for visits to T&CM providers and on purchases of dietary supplements related to pain management (162). It has been demonstrated that out of pocket expenditure on T&CM for supportive care in cancer was significantly associated with increased risk of financial catastrophe and medical impoverishment among upper-middle income countries in South-East Asia (163). Another study demonstrated that job insecurity was associated with less visits to T&CM practitioners (164). Furthermore, it is known that T&CM use is most often attributable to socioeconomic status (SES), i.e., those with a higher SES are more likely to use T&CM than those with a lower SES (165). These findings point to a socioeconomic inequality in possible effective health service use (166).

This lack of reimbursement is undesirable not only from a moral and pragmatic but also from an economic perspective. As was argued earlier in this paper, findings from economic modeling research suggest that combining T&CM and biomedicine may improve cost-effective long-term outcomes (91–95). Therefore, reimbursement of effective T&CM may be better when looking at the whole picture.

Recommendation regarding goal 3:

- Ensure access to T&CM and biomedicine interventions worldwide
- Reimbursement of all healthcare interventions that are safe and effective
- Cost effectiveness research on including T&CM in reimbursement policies, and on the effects of prevention, healthy lifestyle and self-care on healthcare costs.
- Provide information and ensure easy accessible training in effective affordable self-help strategies

### Other recommendations

We have argued that the concept and principles of IM provide concrete healthcare actions and measures that are worth to further implement and investigate in the light of the increasing global health threats such as chronic diseases, pandemics, and ever-increasing healthcare cost. We made suggestions concerning the three main strategies in the 2014–2023 WHO Traditional Medicine strategy. We propose three additional suggestions:

1. Fostering international cooperation between Academic Consortia for Integrative Medicine and Health

There is currently no global platform or organization that represents clinical practice, education, and research on T&CM from the perspective of Western academic medicine.

To facilitate the global integration of T&CM, we therefore propose to establish a Global Matrix of Academic Consortia for Integrative Medicine and Health and the WHO, its global and national collaborative centers, T&CM providers, International society for T&CM research (ISCMR), other stakeholders and patient organizations.

The establishment of a Global Matrix of Academic Consortia was originally proposed by the former head of the WHO’s T&CM Unit (Dr Zhang Qi) a few years ago. Subsequently, the three national academic IM consortia United States, Brazil and the Netherlands elaborated further on the idea and drafted a first outline. This outline was presented to many stakeholders in May of 2022 during the International Congress on Integrative Medicine & Health in Arizona, USA and during the International Congress on Integrative Medicine in Rome in September 2023. Based on their feedback and suggestions the present paper was drafted and finalized. The next step is to establish a Global Matrix (GM) and to formulate its vision, mission, goals, and objectives. Besides, models for collaboration will be developed centered around the GM’s goals and objectives.

The overall goal of our proposed GM is to support the world-wide integration of T&CM by advancing research, academic education, clinical guidelines, policy, and communication on IM, from the perspective of academic medicine. To reach this goal, we foresee the following objectives in the next 5 years:

- To connect academic consortia on T&CM and all other stakeholders and organizations in all six WHO regions: the African Region (AFRO), the Eastern Mediterranean Region (EMRO), the South-East Asia Region (SEARO), the Region of the Americas (AMRO), the Western Pacific Region (WPRO), and the European Region (EURO)
- To support the establishment of academic consortia in the six WHO regions
- To develop and advance a global agenda that sets priorities for research, education, clinical guidelines, policy, funding and communication
- To exchange academic knowledge, implementation experiences and evaluation tools with stakeholder in the field of T&CM
- To provide insight into the evidence status of T&CM, by means of evidence maps of clinical effectiveness in T&CM.
  To provide support and collaborate with the WHO, the development of the WHO Traditional Medicine strategy, its 26 WHO collaborating centers on T&CM, the WHO Global TM Center, the regional offices and other stakeholders such as but not limited to ISCMR and PAHO.

1. An international research agenda

An important step forward to address the challenges regarding the lack of (good quality) evidence for (cost)effectiveness, safety and the conduct and application of T&CM research is to develop an international research agenda based on ‘gaps’ and then address research priorities. This should also include cost-effectiveness studies, as controlling costs is a major challenge in healthcare. Ongoing mapping studies on the clinical effectiveness of T&CM of the BIREME/PAHO, Brazilian Academic Consortium for Integrative Health and the WHO can assist in this research prioritization by identifying the evidence gaps and weaknesses in methodological designs.

The use of AI in generating evidence could be considered, as there are many research questions and a shortage of people and funds.

Besides (cost) effectiveness of T&CM, there is also a need to focus on implementation science, as there seems to be a gap between the existing evidence and current practices, especially in academic health centers.

1. International policy and guidelines

The status of integration of T&CM varies considerable between member states and depends mainly on personal philosophies, values and perspectives of regulators, clinicians, and practitioners in each country. We recommend that the main challenges regarding quality assurance, safety, proper use, effectiveness, and integration of T&CM as identified in this paper, are addressed in an updated or new WHO Traditional Medicine strategy. However, to tackle these challenges and further the field, we propose that a new policy (strategy) of the WHO is closely linked to the clinical, academic and research practice of T&CM. We are of the opinion that a direct and dynamic interaction between policy, research and practice will support, facilitate, and accelerate the integration of biomedicine and T&CM. To do so a worldwide network of cooperating (consortia of) academic healthcare centers and researchers is needed. A Global Matrix may be the best way to facilitates this, as it connects all involved in T&CM while at the same time ensuring everyone’s autonomy.

## Conclusion

This paper offers an overview of integrative medicine approaches and offers recommendations for the upcoming update of the present WHO strategy on T&CM from the perspective of academic medicine. With this we hope to contribute to an integrated, compassionate, person-centered global healthcare in which anyone, regardless of their illness, sociodemographic or cultural background, worldview or treatment preference can receive the care he or she needs and is most in line with their values.

Our proposal of the establishment of a Global Matrix should not be seen as an endpoint, but rather as a start. We are aware of many organizations and societies with similar goals; however, our proposal is based on the perspective of academic medicine, which is lacking. We invite global dialog with stakeholders on this proposal and critical issues being put forward.

## Author contributions

RH: Conceptualization, Writing – original draft, Writing – review & editing. RG: Writing – review & editing. CP: Writing – review & editing. SS: Writing – review & editing. AL: Writing – review & editing. HC: Writing – review & editing. DG-P: Writing – review & editing. MJ: Supervision, Writing – review & editing.

## Footnotes

^1^T&CM is now also sometimes referred to as TCIM.

^2^www.lifestylemedicine.org/resources/Documents/LifestyleMedicine-LiteratureReview.pdf

^3^https://boletin.bireme.org/en/2017/11/29/cooperation-to-strengthen-traditional-medicine-and-complementary-therapies/

^4^https://www.oecd.org/health/health-expenditure.htm#:~:text=There%20was%20a%20rebound%20in,funding%20to%20tackle%20the%20pandemic.

^5^https://imconsortium.org/member-listing/

^6^http://www.imconsortium.org/about/home.html

^7^https://www.cizg.nl/home/
